# Generating Bulk-Scale Ordered Optical Materials Using Shear-Assembly in Viscoelastic Media

**DOI:** 10.3390/ma10070688

**Published:** 2017-06-22

**Authors:** Chris E. Finlayson, Jeremy J. Baumberg

**Affiliations:** 1 Department of Physics, Prifysgol Aberystwyth University, Aberystwyth, Wales SY23 3BZ, UK; 2 Cavendish Laboratory, University of Cambridge, Cambridge CB3 0HE, UK; jjb12@cam.ac.uk

**Keywords:** Nanoassembly, photonic crystals, polymers, viscoelastic media, shear processing

## Abstract

We review recent advances in the generation of photonics materials over large areas and volumes, using the paradigm of shear-induced ordering of composite polymer nanoparticles. The hard-core/soft-shell design of these particles produces quasi-solid “gum-like” media, with a viscoelastic ensemble response to applied shear, in marked contrast to the behavior seen in colloidal and granular systems. Applying an oscillatory shearing method to sub-micron spherical nanoparticles gives elastomeric photonic crystals (or “polymer opals”) with intense tunable structural color. The further engineering of this shear-ordering using a controllable “roll-to-roll” process known as Bending Induced Oscillatory Shear (BIOS), together with the interchangeable nature of the base composite particles, opens potentially transformative possibilities for mass manufacture of nano-ordered materials, including advances in optical materials, photonics, and metamaterials/plasmonics.

## 1. Introduction

Systematically ordered 3D photonic structures, creating iridescence originating from Bragg reflections of visible light can be found in opal gemstones, butterfly wings, flower petals, and beetles [1,2,3]. Such microstructures are an important subset of 3D photonic crystals; wavelength-scale periodic dielectric structures with the ability to modify the spatial, spectral, and temporal properties of absorbed, reflected, and transmitted light [4], giving distinguishing optical properties (e.g., structural colour), which are not accessible using dyes or pigments. Whilst these effects may be replicated to a degree on 2D surfaces using (e.g.,) imprinting or holography, genuine 3D bulk structures have been notably very difficult to generate artificially on a large scale using techniques that are cost effective enough to allow their widespread use.

In this review, we summarize our recent work illustrating the paradigm of shear-induced ordering of composite polymer nanoparticles, in the generation of opaline photonics materials over large areas and volumes. A particular focus is on consolidating an understanding of the mechanisms of the order–disorder transition of particles in this viscoelastic medium under oscillatory shear, and also the correlations between these mechanisms and the final permanent opaline structures. 

### 1.1. Overview of Synthetic Opal Production

Conventional strategies for engineering bulk-ordered optical materials, such as those for application in solar cells and enhanced photoconductivity [5], have relied upon self-assembly of high and low refractive index components. However, the methods cannot generally be reproduced in any scalable fashion, and the resultant structures also critically lack the mechanical robustness needed for many practical applications. Research into the synthesis and characterisation of opaline materials is an active and wide field [3,6]; here, we present an overview of some of these methods.

The earliest attempts at artificial generation of periodic structural color included etching and “direct-write” approaches on bulk materials and coatings [7]. More elaborate “top–down” strategies have since evolved, including various methods of lithography and lithographic patterning [6]. The bulk properties of functional materials may also be designed, such that meso- and micro-scale ordering may develop as a result of external stimuli and/or phase transitions of the system. Examples include the behavior of elastomers and polymer networks [6,8], or the elastic instabilities that have been studied in soft layered composites [9]. 

“Bottom–up” approaches have been spearheaded by the use of colloidal particles (e.g., solution based silica beads and polymer microspheres) in thermodynamically driven self–assembly towards artificial opals and photonic crystals; an approach which is widely studied over many years [10,11,12,13]. Whilst impressively well-ordered opals may be generated by relatively slow sedimentation methods or under the action of shearing, the resultant structures are only tractable over small volumes and areas, and cannot generate permanent stable structures in any large-scale fashion. Ancillary methods, such as stabilization of the ordering or alignment of colloidal particles in electric or magnetic fields [14,15,16], are also reported. As an illustration of possible applications in this area, post-alignment curing methods have been used to generate printed structural color in this fashion [17].

As an important related exemplar to the progress with “polymer opals” reviewed in this paper, the group of Gallei et al. in particular have illustrated the possibilities of using melt-shearing in microporous ensembles to generate ordered optical structures in soft matter. Additionally, the concept of using hybrid starting-components, such as core–shell particles, to generate dielectric periodicity is demonstrated here [18,19]. The uses of further engineered soft-matter architectures, such as colloidal “brush” particles and polymer-tethered colloids, for use in hypersonic phononics at micron length scales are also reported [20]. A recent example of bio-inspired approaches to structural color generation comes from Fu et al., where hydrogels assume ordered mesoscale formations, including inverse opaline scaffolds, with the additional abilities of environmental survivability and self-repair [21].

### 1.2. Shear-Ordering in Viscoelastic Media

**Figure 1:**
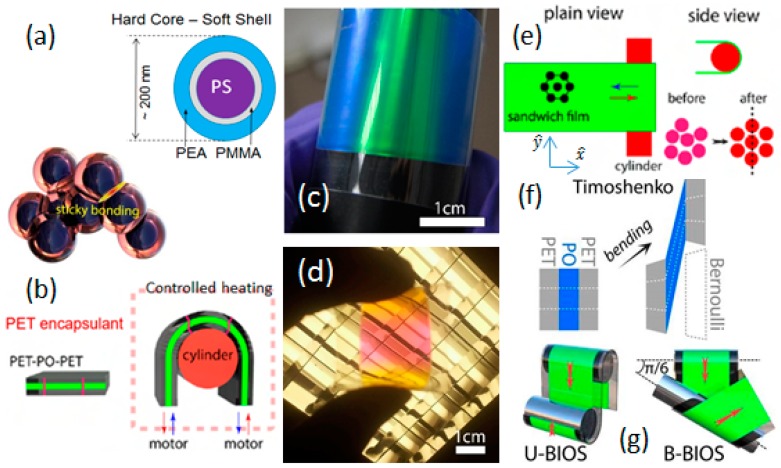
(**a**) Core–shell architecture of precursor particles for viscoelastic assembly, showing the hard-core (polystyrene, PS), grafting layer (poly-methyl methacrylate, PMMA) and soft-shell (poly-ethylacrylate, PEA) components, which have a short-range “sticky bonding” interaction; (**b**) illustrates the mechanics of Bending-induced Oscillatory Shear (BIOS), with the polymer opal (PO) thin-film layer encapsulated by rigid polyethylene terephthalate (PET) tapes; (**c**,**d**) show photographic images of the resultant polymer opal films in reflected and transmitted white light respectively; (**e**) illustrates the principle of ensemble ordering as an oscillatory bending shear is applied; (**f**) Mechanism of BIOS inside the PET–PO–PET “Timoshenko sandwich”, with the dashed white lines indicating the bending-induced deformation in different layers; (**g**) shows the geometry and shear-processing direction of uniaxial U-BIOS (left) and biaxial B-BIOS (right) as indicated by arrows, in consistency with the conventions shown in (**b**,**e**). (Adapted from Ref. [28] with permission of Nature Publishing Group).

By marked contrast with the examples described above, recent work has demonstrated how synthetic opals (or “polymer opals”), based on permanently shear-assembled arrays of composite polymer microparticles, are a promising platform for next generation manufacturable photonics materials, flexible coatings, fibres, and sensors [22,23,24,25]. These particles (diameters ~200–300 nm) are constructed of bulk-produced rigid polystyrene spheres, onto which a softer polyacrylate (polyethylacrylate, PEA) shell is grafted, providing a “gum-like” matrix. As illustrated in Figure 1a, the PEA is bonded to each polystyrene (PS) sphere as a shell and is viscoelastic above its glass transition temperature T_g_ ≈ 0 °C with η = 10^3^–10^4^ Pa·s at 100 °C. Mechanically, these assembled composites behave like rubbers, with commensurate stretch- and bend-tuneability of structural color [26,27].

The development of these principles of nanocomposite shear-assembly into a controllable “roll-to-roll”-type process, known as Bending-Induced Oscillatory Shear (BIOS) [28], forms the focus for this review, as we summarize the key methodology, phenomenological results, structure determination, and preliminary physical models. The film/lamina structures generated are typical up to 1000 crystalline monolayers thick, and the nature of processing is readily compatible with bulk-scale sample production, as well as many ubiquitous methods in polymer processing.

In terms of fundamental rheology, a key clarification has been that the systems of colloidal suspensions widely reported in the “bottom–up” generation of ordered materials [11,29,30] cannot be directly compared to the viscoelastic media of polymer opals. The core–shell design of particles means that, even at the point of melting, the polymer opals do not contain a discrete fluid phase. Commensurately, the system is athermal with no diffusive Brownian motion and therefore no ability to self-assemble; the mobility of the spheres is highly inhibited by the strong dissipation inside the system due to the viscoelasticity of the PEA. The colloidal self-assembly systems are entropy driven and metastable equilibrium structures are determined by a resultant potential energy minimum from a combination of dispersive forces, interparticle collisions, and electrostatic forces, etc. [31]. By fundamental contrast, the equilibrium in polymer opals is mainly associated with the accumulation and release mechanisms of strain energy from external forces, e.g., shearing, stretching, particle–particle torque etc. These mechanistic differences are highlighted by the relatively large strain magnitudes (of γ ~ 300%, well beyond the material yield-strain) at which the efficacy of the shear-ordering is optimized; again, in marked contrast to oscillatory shear-assembly in low-viscosity colloids, where typically γ < 100% [32,33,34]. The nature of the shear-assembly process in such viscoelastic media has also been shown to greatly reduce the requirements for particle polydispersity in crystalline ordering [35], with assembly still possible with disorder levels far beyond that feasible for colloidal self-assembly.

There have been no previous reports in the literature, which would provide any pertinent model for how the sort of massive scale assembly evident from the structure color in Figure 1c,d can occur, despite its utility in creating very large-scale ordered nanostructures and represents a step change away from the monolithic architectures currently relied upon. Here, we review our preliminary attempts at intuitive microscopic modelling of nanoparticles in such viscoelastic media under bulk-shearing, linking in with key experimental and phenomenological findings. The model indicates how the reduction in shear viscosity with increasing order of each layer produces a crystallization front flowing from the surfaces and a saturated crystalline ordering behind it, strongly concurring with the structural characterisation of samples from diffractometry and electron microscopy studies.

## 2. Overview of Recent Studies

### 2.1. Processing Methods; Bending-Induced Oscillatory Shear 

The archetypal polymer opal (PO) materials used in viscoelastic shear assembly are based on ensembles of spherical core–interlayer–shell particles, as illustrated in Figure 1. These particles are synthesized using a batch multi-stage emulsion polymerization process and freeze-dried, as previously reported [36,37]. The particle precursors range in size from 180 nm to 260 nm in outer diameter, and consist of a rigid heavily cross-linked PS core, coated with a polymethacrylate-based (usually poly-methyl methacrylate, PMMA) grafting layer, and a soft poly-ethylacrylate (PEA) outer shell. The net refractive index contrast (Δn) between core and shell materials in this configuration is ≈0.11, and the volume fraction of cores ≈ 55%.

The procedures for polymer opal thin-film sample production have been reported at length in several of our earlier publications [38,39,40], and we provide a brief methodological summary here. As synthesized precursor particles are mixed and recirculated in a polymer extruder fitted with a rectangular die and therefore squeezed into ribbons. As per standard protocol, the ribbons from extrusion are then rolled into thin films with a standard thickness of ≈80 μm and laminated between two rigid polyethylene terephthalate (PET) sheets of high Young’s modulus. The BIOS process is then applied to the sandwich structure immediately after the continuous rolling lamination, and an ordered opaline layer is obtained by the repeated application of oscillatory shearing, as illustrated in Figure 1b [28]. Effectively, the BIOS processing is achieved by mechanically oscillating (cycling) the sandwich structure under tension around a fixed cylindrical surface (typically of diameter ~12 mm) at a stabilized temperature of 100 °C. Since the PET encapsulant film is far more rigid than the PO, the overall PET–PO–PET laminate may be considered as a Timoshenko sandwich beam [41] as illustrated in Figure 1f. By bending the laminate around a cylinder, strong shear is created inside the PO purely parallel to the surface, thus generating strains of magnitude up to 300%. This process may additionally be seen as a more controllable, reproducible extension of the Edge-induced Rotational Shearing (or EIRS) method of earlier reports (Figure 1b) [39].

As a further innovation, two variants of the BIOS process have been employed, in which either the shear direction through the rollers is maintained as unidirectional (U-BIOS), or we provide a bidirectional roller alignment (B-BIOS) where it is first sheared along the direction perpendicular to the roller axis and then along a 30° twisted direction (Figure 1g). This allows further investigation of the effects of shear direction ($\hat{x}$ in Figure 1e) on the anisotropy of ordering.

### 2.2. Rheometry and Time-Dependance of Shear Ordering

While the phenomenon of shear-induced ordering in viscoelastic polymer opals had been clearly demonstrated since early reports [42,43], relatively little was initially known or understood concerning the detailed mechanism or time dependence of crystal formation [44].

**Figure 2:**
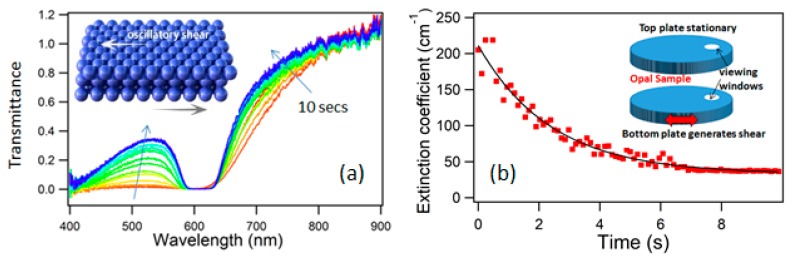
(**a**) Optical spectra taken during the crystallization of an opal sample by means of an oscillatory shear (inset), showing the progress of crystallization over 10 seconds (oscillation frequency of 1 Hz, strain amplitude 200%, shear rate 2 s^−1^); (**b**) Optical extinction coefficient at λ = 530 nm as a function of shearing time, together with a fit to an offset exponential function, of lifetime τ = 2.4 s. The inset illustrates the experimental geometry used in shear-cell measurements, with the opal sample material introduced to fill the entire space between two plates with optical viewing windows. The parallel plate spacing is 300 μm, with a total cell volume of ~0.2 μL. (Adapted from Ref. [44] with permission of American Physical Society).

Using a shear-cell rheometer (manufacturer LINKAM, Surrey, UK; model CSS450) equipped with optical windows, to allow simultaneous interrogation of structural color due to crystallization, the batch composite particles are studied under oscillatory shear (standard cycle frequency of 1 Hz, shear rate 2 s^−1^). To achieve the necessary viscoelastic response, the cell was heated to a fixed temperature of 100 °C, and a cyclical strain amplitude of 200% applied. As seen in Figure 2a, as shear oscillations progress a clear and intense resonant “stopband” develops in the spectrum of transmitted light. The increasing Quality (Q)-factor of the resonance and the commensurate change in transmission at the low-wavelength side of the resonance is directly indicative of the change in the particle spacing (radial distribution function) in the crystal. Hence, characterizing this short-wavelength end (around λ = 530 nm) enables us to directly correlate spectral changes to increased or decreased ordering, and an analysis of how this shear-ordering of the opaline crystal progresses as a function of shearing time is shown in Figure 2b. Under these conditions of crystallization, a timescale for the disorder–order transition of τ = 2.4 sec is extracted. Empirically, at low strain magnitude values below 100% there is relatively little decrease of the low-λ optical extinction with time, indicating a limited suboptimal development of crystallization. Strains in the range of ~100–250%, however, produce much greater changes in the extinction coefficient, implying crystallization processes which are equilibrated or near complete. As the strain magnitude increases towards 400%, a significant rise then occurs in the low-λ extinction, indicating an above-optimal strain, possibly associated with “shear-melting”-like processes [45], and a limited development of crystallization.

A key outcome of this study, and a basis for future theoretical models, was a confirmation that ordering progresses *monotonically* with increasing application of oscillatory shearing of suitable amplitude. Critically, the periodicity required for the production of structural color is not disrupted during the cyclicity of processing. Indeed, physical intuition suggests that the optical signatures of ordering will not proceed indefinitely, but will reach an asymptotic limit at a time where crystallization has completed. Furthermore, the expectation is that as the crystalline ordering increases, it will become more susceptible to damage by further motion. There should therefore be an equilibrium point at which the rate of damage (increasing defects) is equal to the rate of crystallization. This expected behavior is strongly verified, as illustrated in Figure 2b.

### 2.3. Optical Properties and Crystalline Ordering

In addition to a clear demonstration of large-scale assembly, the reproducible high-quality samples made possible with the BIOS method facilitated detailed structural studies and, crucially, the first direct experimental determinations of the detailed PO structure. New physical insights are also gained into the microscopic mechanisms behind crystallization of this rheologically unique system, via small-angle X-ray scattering (SAXS) and focused ion beam–scanning electron microscopy (FIB–SEM) microscopy of a variety of samples produced under different BIOS parameters.

**Figure 3:**
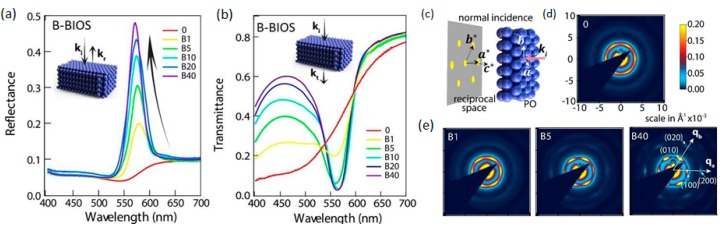
(**a**) Reflectance spectra of B-BIOS samples, with number of shear oscillations indicated in the caption; (**b**) corresponding transmittance spectra of B-BIOS samples; (**c**) Illustration of small-angle X-ray scattering (SAXS) at normal incidence with incident wave vector *k*_i_, unit vectors ***a***, ***b*** of hexagonal lattice, a*, b* and c* are corresponding reciprocal lattice unit vectors; (**d**) SAXS pattern of samples prior to BIOS at normal incidence, with q_a,b_ along a*, b*, respectively; (**e**) shows corresponding SAXS patterns of B-BIOS samples. All SAXS images are (10 × 10) × 10^−3^ Å and on the same colour scale. (Adapted from Ref. [28] with permission of Nature Publishing Group).

Ordered PO films of thickness ~100 μm are obtained after the BIOS process, exhibiting bright structural colors, which are vividly observable in both spectral reflectance and transmittance (Figure 3a,b) from Bragg diffraction at wavelength, $\lambda =2d_{hkl}{\left({\bar{n}}^{2}-sin^{2}\theta \right)}^{1/2}$, where *d_hkl_* is the plane spacing, $\bar{n}$ = 1.52 is the mean refractive index for POs, and θ is the incident angle. As is expected from earlier characterisation described above, the bulk crystalline ordering as inferred from structural color intensity very markedly increases with repeated application cycles of the BIOS process. From SAXS characterisation of films (Figure 3c–e), the spheres are found to be packed in hcp planes, with the most close-packed direction parallel to the BIOS processing direction $\hat{x}$ (Figure 1b,e,f). The BIOS effect was again found to depend on the parameters of shear strain γ, shear rate $\dot{\gamma }$, and temperature. These studies also demonstrated how the degree of ordering increases incrementally as BIOS is applied; the pre-sheared sample in Figure 3d shows a ring-like SAXS pattern indicative of a poorly ordered amorphous structure with only vague signatures of a six-fold diffraction spot symmetry. However, as the number of BIOS cycles is increased, the spots associated with the random-hcp (r-hcp) packing symmetry develop into well-defined features, with separation between scattering peaks corresponding to layer spacing of 197 nm in real space (or 5.1 μm^−1^ in reciprocal space). As the number of BIOS cycles increases to ~40 and beyond, the progression of crystalline ordering brings signatures of higher spatial frequencies (e.g., the {2,0,0} crystallographic planes) into view.

**Figure 4:**
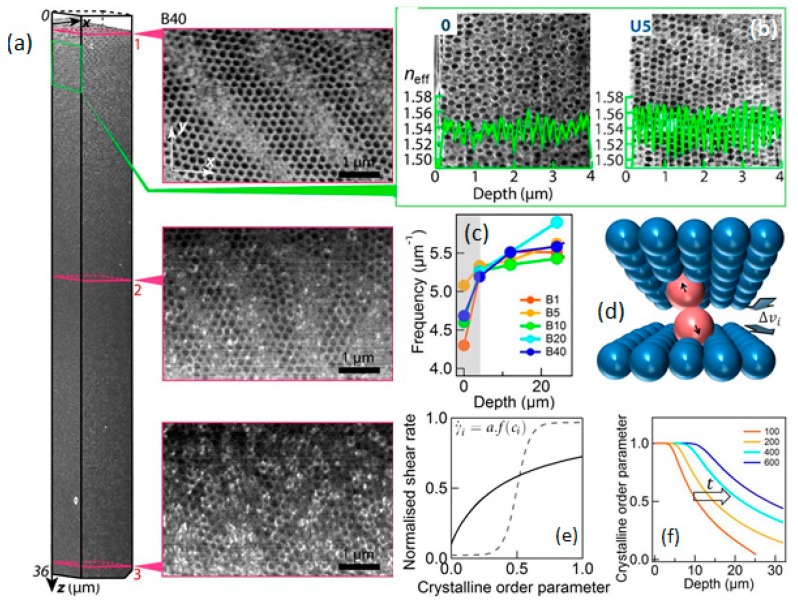
(**a**) Reconstructed 3D structure of PO sample after 40 oscillations of B-BIOS with horizontal slices extracted at depths labelled. Cuts are slightly tilted to the hcp planes, giving images of several neighbouring layers separated by blurred stripes; (**b**) Cross-section scanning electron microscopy (SEM) images of samples pre-BIOS (0), and after 5 oscillations of U-BIOS around different depths. Extracted *n*_eff_(z) variations (left axis) are superimposed (green); In parts (**a**,**b**), samples are pre-stained with RuO_4_ to enhance the electron density contrast. Slices in xy planes normal to the sample surface are successively cut with focused ion beam (FIB), and imaged by in situ SEM. (**c**) Main spatial frequencies of *n*_eff_ at different depths in B-BIOS samples; (**d**) illustrative model of ordering forces for individual spheres out of position, with interlayer relative velocity Δ*v*_i_. (**e**) Nonlinear transfer relation between normalized shear rate and crystalline order in each layer, for polymer opals (solid) and colloids (dashed). An order parameter of 0 represents disorder and 1 corresponding to perfect crystalline ordering; (**f**) Resulting crystalline order versus depth for increasing total strain applied (red to blue). (Adapted from Ref. [28] with permission of Nature Publishing Group).

As a complementary structural characterisation method, Figure 4 shows scanning electron microscopy (SEM) of BIOS produced samples, where cross-sections of the opaline structure have been extracted by focused ion beam (FIB) sectioning. The 3D equilibrium structure of the sample with 40 passes of B-BIOS (B40) may be reconstructed by the stacking of many slices (Figure 4a) allowing the in-plane organization parallel to the sample surface to be extracted at different depths. Near the surface, spheres pack in hcp planes with the close-packed spheres along the BIOS processing direction $\hat{x}$; some multi-domains are also observed along with numerous dislocations. The packing order of the hcp planes degrades with depth, and the layers finally break into islands of hcp fragments at z > 30 μm. After 40 passes, around 200 well-ordered hcp layers may be observed, which theoretically should produce ~90% Bragg reflection at normal incidence, a significant fraction of this light is scattered into a wider angular cone by dislocations [39,46]. Reconstructing cross-sections for different samples at different depths (Figure 4b) and extracting the sphere positions allows the effective refractive index *n*_eff_(z) distribution with depth to be calculated. Both the main spatial frequency (Figure 4c) and the refractive contrast between PS and PEA components Δ*n*_eff_ reveal details of the BIOS mechanism. The initially disordered arrangement rapidly crystallizes close to the surface, but with a spatial frequency which is some 15% smaller in the surface layers compared with the bulk, emphasizing the role of the nonlinear shear strain in achieving ordering at different depths throughout the film thickness. Overall, the observed variation in ordering with depth and wide crystallization front as BIOS proceeds, presents behaviours very uncharacteristic of either solution-based colloids or gravity mediated granular systems, emphasizing the need for new theoretical approaches, as described in the following section.

The analyses and comparison of the U-BIOS and B-BIOS processing methods (Figure 1g) show the effects of shear direction on the anisotropy of ordering, as the direction of shear orients the close-packed lines of spheres in each hcp plane controlling the in-plane ordering [27,28]. Detailed analysis of anisotropies in SAXS show that, while B-BIOS induces better positional order, U-BIOS induces better orientational order than B-BIOS; U-BIOS creates more line dislocations with Burgers vector transverse to the processing direction ($\hat{y}$ in Figure 1e). These persisting line defects for U-BIOS restrict shear thinning, and can only be annealed out through line defect annihilation by forces along $\hat{y}$, which are absent without B-BIOS. This leads to less surface screening in U-BIOS and better ordering in the bulk. 

As a final point of note here, we consider the implications of these, and previous, findings for our understanding of the nature of crystalline defects in the as-generated “polymer opal” structures. In previous studies, crystallographic evidence of stacking faults and/or twinning within the cubic structures was presented, with these faults introducing a certain level of disorder distributed throughout the films [39,43]. It is likely that these characteristics are also intrinsic to many self-assembled periodic structures, in the absence of direct next–nearest neighbour interactions, including those found in nature. The microstructural observations of orientational order and line defects described above are also of interest in the context of the earlier observation of directional chaining effects within the polymer opal films, and the resultant peculiar angle-dependence of the light scattering cone [46,47]. As a first order indication of the presence of lateral fluctuations in the alignment of parallel chains in each layer within these photonic crystals; analysis of the lateral broadenings of these cones in reciprocal space show correspondence to real-space dimensions of 3–5 μm, implying lateral defects of size around 10× the crystal layer spacing [48].

### 2.4. Theoretical Framework

Whilst theoretical models are reported for oscillatory shear-ordering in colloidal systems [32,49,50], they are completely outside of the range of rheological applicability here. Indeed, this process and the unique core–shell system has already generated the largest regular nano-assembled structures ever demonstrated up to the km-scale. We now review preliminary success with intuitive rheological ordering models, which deliver an improved understanding of the exceptional ability of BIOS for inducing order in solvent-free viscoelastic systems of particles [28].

The application of shearing in BIOS to the PET–polymer opal–PET Timoshenko sandwich system (Figure 1e) may be effectively modelled with mesoscale mechanical simulations using Abaqus finite element analysis software [51]). This shows how the 180° bend of the sandwich around the rollers depicted generates strong shear is inside the PO film purely parallel to the surface; this gives strain γ increasing up to 300% in accordance with experimental measurements. Our combined microscopic characterisation (Figure 3 and Figure 4) imply that ordering into hcp planes proceeds progressively inwards from the flat film surfaces. A direct comparison of optical and SAXS measurements show that stacking plane and in-plane order both improve at the same rate, indicating that they arise from a single complementary process. However, contrary to the case of diffusing colloids in solution, which show sharp crystal interfaces [52], here we find a gradation of ordering. Previous measurements show shear thinning in this composite system with increasing order [40,42] and since diffusion is virtually absent (Péclet number, Pe > 10^6^), we therefore infer that it is order-dependent shear viscosities that underpin the driving mechanism. As the disordered spheres separate into distinct planes, the shear viscosity drops (Figure 4d). At the same time, the viscosity also reduces when in-plane ordering leads to neighboring rows of spheres aligned along the shearing direction. A similarity to the focusing of forces in ‘jamming’ phenomena is noted [53], whereby displaced defect spheres will experience strong restoring forces pushing them into ordered positions (Figure 4d). 

To parameterize this shear thinning, we define a local shear rate $\dot{\gamma }$ for each layer *i*, which increases as the local crystalline order parameter *c*_i_ increases (Figure 4e inset); *c*_i_ is an archetypical direction-averaged order parameter varying from 0 to 1 (disordered to perfect order, respectively), while *a* is proportional to the total shear strain applied across the entire film thickness. An increasing shear strain (as the films are bent around each roller) is distributed non-uniformly between different layers depending on their local order *c*_i_ (*z*_i_,*t*) at depth *z*_i_ and time *t*. According to Newton’s 2nd law, additional velocity (∝ shear rate) added to each layer generates an interlayer drag force proportional to the velocity difference between neighboring layers, i.e., $F_{i}dt\propto \left(v_{i}-v_{i-1}\right)\propto \partial_{z}{\dot{\gamma }}_{i}$, which incrementally improves the local ordering as described above. By numerical integration, we obtain spatio-temporal solutions, *c*_i_ (*z*_i_,*t*), for the development of ordering under applied shear within the sample. As a simple generic model that encapsulates the crucial elements, depending on the transfer function *f*(*c*) different behaviours are obtained, reconciling the behavior of a growth front and the sharp threshold between ordered and amorphous regions seen in colloidal systems (Figure 4e), with a more gradual shear thinning in the viscoelastic medium which strongly resemble that found experimentally in polymer opals. In particular, the model predicts a long-tailed crystallization front flowing from the interface, producing a saturated crystalline order behind it (Figure 4f). Increasing order in the surface layers reduces their viscosity, resulting in strains within the bulk interior being progressively screened out by the easily sheared crystallized surface. Improving deep order thus requires much higher strains, and eventually shear ordering fronts from top and bottom surfaces meet in the film center. Ordering in this system thus relies on irreversible dissipative forces focused on out-of-place spheres; forces which are furthermore independent of the sign of the velocity difference between neighboring planes. Overall, in contrast to steady-state shearing which pulls the crystallites apart as it is increased, oscillatory shear is able to repeatedly nudge errant nanoparticles towards the lowest viscosity state, again in strong corroboration with the experimentally observed behaviours.

### 2.5. Nanoassembly of Polydisperse Particles; Polymer Opal “Alloys”

Emulsion polymerisation, which we use in synthesis of core–shell particles for viscoelastic shear-assembly, is well established for sub-µm particles with polydispersity of 1% or better [37]. These (low) levels of polydispersity are usually sustainable in other self-assembling crystalline systems, such as flow ordered colloidal suspensions; however, crystallization is suppressed by a critical level of polydispersity, typically around a few %, above which crystalline ordering is strongly suppressed [12].

In a recent paper on BIOS assembly in opaline alloys [35], we show how visco-elastic nanoparticle assembly has much lower requirements for monodispersity (by an order of magnitude) than any previous technique, and that polydispersity is thus not the limiting factor in ordering. Additionally, it may be considered how the size ratio between different components is a critical parameter in the generation of colloidal alloys. In particular, colloidal alloys with a diameter ratio between the small and large components (D_S/L_) of less than ≈0.3 or >0.6 are very rare, irrespective of the method of assembly used, so that a D_S/L_ of ≈0.5 is energetically required for any degree of success in alloying [54]. The BIOS method, however, facilitates the generation of polymer opal alloys comprised of spheres with size ratio D_S/L_ = 0.71–0.85, in both ordered binary and ternary configurations. Layered structures are observed to form parallel to the sample surface, with a spacing which shows an exponential variation with depth, and dominated (but not exclusively set by) the largest sphere. Layered structures are observed to form parallel to the sample surface, with spheres inside the layers show a dominant hexagonal packing, in a behavior very similar to single-component POs, and with the most closely packed direction parallel to the shearing direction. 

**Figure 5:**
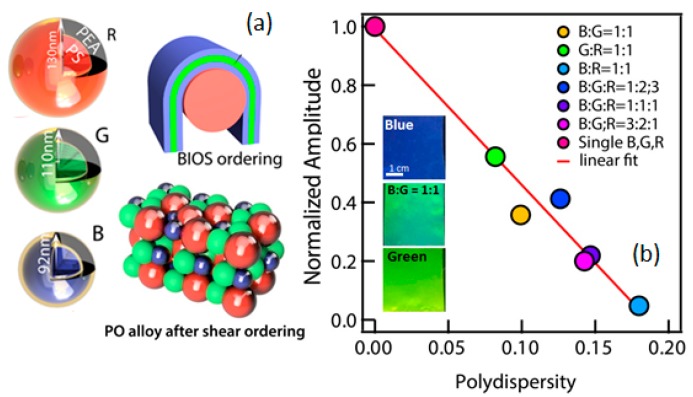
(**a**) B, G, and R represent core–shells spheres of different sizes that make blue, green, and red single-component POs, respectively, and which may be BIOS processed to produce opaline alloys; (**b**) Normalized Bragg peak amplitude versus sample polydispersity; the colored points denote the various binary and ternary alloys, as per the figure legend. The inset shows photographs of BIOS-processed POs with different blue (B) and green (G) components, including a 1:1 alloy with intense structural color. (Adapted from Ref. [35] with permission of Wiley).

In Figure 5a, the differently sized core–shell components used in alloy production are illustrated, with highly monodisperse “red”, “green”, and “blue” elements being co-processed into genuine polydisperse alloy structures with random size-occupancy of each lattice site; all the available experimental evidence indicates that there is no site correlation or gross separation of sizes, occurring in these systems. In Figure 5b, we can see how alloy samples with strong structural color as readily apparent to the naked eye, are generated via BIOS. The hue/saturation of the colors of the PO alloys varies with the number ratios between different components, as confirmed by the measured optical reflectivities at normal incidence and Bragg peaks have lower amplitude than those from the single-component samples (Figure 5b). Somewhat surprisingly, however, the spectral positions of the peaks from the PO alloys are found not to simply be the mean of their sub-component peak wavelength positions (i.e., λ_B_, λ_G_, λ_R_). The peaks of the alloys are always shifted to longer wavelengths than the corresponding weighted average, which is attributed to the spacing between ordered layers in the alloys being more strongly influenced by the larger component spheres. 

The standard concept of polydispersity index (*PDI*) is applied in order to quantify the magnitude of particle size variation of spheres in the alloys; $PDI\;=\sigma /D_{0}$, where σ is the standard deviation and *D*_0_ is the mean diameter of the spheres. A direct correlation between the normalized amplitude of the PO alloys with polydispersity is empirically observed (Figure 5b). The reflection amplitude drops with increasing *PDI*, with extrapolation indicating that no structural resonant peak exists for *PDI* > 20%, thus setting an upper tolerance limit of particle (bimodal or tri-modal) polydispersity for BIOS-generated ordered PO alloys. To place this into context, this is around a four-fold improvement as compared to the 5% polydispersity found to destroy/suppress all crystalline ordering in colloidal suspensions [55]. 

## 3. Discussion

Aside from the putative interests into novel application of “polymer opals” [56], there are several key points of fundamental interest that have arisen from the recent studies into shear-assembly of nanocomposite in visco-elastic media reviewed here, which we now aim to summarize. 

Indeed, compared with granular systems and colloids where crystals are obtained with γ < 100%, and often below the strain needed for plane-slippage [32,57], crystallization of POs demands larger γ because sufficient shear stress is needed to shift particles well beyond the yield strain to facilitate their repositioning. This may be further elucidated in terms of the specific microscopic forces between particles in the case of POs; the elastic nature of the shells dictate that contact forces between adjacent particles are extremely high and it is unknown to what extent forces due to chain entanglement between the PEA shells might be generating rolling of the particles (this is not yet considered in the models). Therefore, small movements associated with relatively small strains are accommodated by the elastic shell, without resulting in any permanent repositioning of the shell–shell boundaries. Above a critical strain (and shear), however, these frictional forces between shells are overcome. As corroborated by both experiment and modelling, there is a monotonic relationship of the progress of induced ordering and crystallinity to shearing cyclicity, resulting in permanent end structures. In both the shear-cell and thin-film (i.e., BIOS) systems studied, the final samples are then stable on timescales of years.

Studies have now demonstrated the ability of the BIOS method in creating new equilibrium structures which are not able to develop in systems of colloidal self-assembly, and in inducing crystallization in a diverse range of polydisperse PO systems [58]. As a significant potential breakthrough, large-scale flexible photonic crystal films, potentially incorporating a wide range of engineered polymeric composite particles, can thus be made with much less stringent requirements for monodispersity.

Future directions might include using BIOS to generate opaline arrays of anisotropic nanoparticles [14] with stretch-tunable birefringence, or scaling to much larger particles to access infrared coating applications. In terms of the new and generic model of order-dependent shear-viscosity that has been developed for BIOS, it may be noted that first principles calculations or direct measurements of this transfer function *f*(*c*) (see Figure 4e) are of great interest for quantitative prediction, especially given the many different conceivable sizes and shapes of nanoparticles [59]. In addition to the instructive distinctions from colloidal crystals described here, in terms of the spatio-temporal behavior of disorder–order transitions, we can anticipate that there may be further application and development of this intuitive model to other crystalline systems in materials science.

## 4. Conclusions and Outlook

Recent research into shear-assembly of nanoparticles in visco-elastic media illustrates completely new paradigms in addressing the long-standing challenges for generating bulk-scale photonics materials and functional materials with ordering on the sub-micron length scale. Whilst it has been long established that other competing routes towards nano-ordered materials, such as colloidal or discotic self-assembly, may show a suitable degree of crystalline ordering of particles, it is both the lack of mechanical robustness and any reliable scalability, which are key differentiators here. As also reviewed here, a potentially significant progress has also been made with ensemble *polydispersity*, with this no longer being a key limiting factor in nanoparticle ordering, as with many earlier methods of self-assembly. Emulsion polymerisation, generating sub-µm particles with polydispersity of 1% or better [37,60], has now been routinely used in the synthesis of bespoke new core–shell composites. 

In terms of future applications, the extension of this archetypal core–shell polymer opal system into more highly engineered smart materials may be actively pursued; for example, composite metal-dielectric opals, opals constructed from anisotropic arrays of particles, or opals with a thermochromic (colour changing with temperature) effects. The application of soft-matter photonic structures to sensing and detection has also attracted a great deal of scientific interest, and the proven adaptation of polymer opals to a variety of different chemical, electrical, mechanical and light stimuli makes them a prime candidate system for further research [61].

However, the progress in generating “polymer opals” based on nanospheres, as reviewed here, is only the first step. The experimental evidence points strongly to the rheology of the core particles being governed by the viscoelastic nature of the PEA shell quasi-matrix; hence, these fully scalable methods and principles should be applicable to more complex architectures on such small length scales, with applications to bio-mimetics and optical materials. This is in addition to a host of new scientific insights into tunable analogue structures, which are mechanically impossible in more conventional “monolithic” photonic structures. On a similar note, the development of higher Δ*n* opaline structures, by the integration of dielectric components or high-*n* polymers [37] will potentially bring access to many of the advanced functionalities associated with photonic crystals [4,62], namely strong optical confinement/localization [63], slow-light [64] and non-linear response, within the platform of large area BIOS generated structures.

As plastics become increasingly important as functional materials, sustainable routes are also critical, and a benefit of the general BIOS processing methods proposed here is its wide applicability to green-produced polymers, not restricted to any specific polymer. Future work in this area will thus aim to generate new large-scale photonics platforms, and facilitate fundamental experimental studies of the physics of photonic crystals, structural colour, and the self-assembled optical structures seen in Nature.
